# Phylogenomics of Southern European Taxa in the *Ranunculus auricomus* Species Complex: The Apple Doesn’t Fall Far from the Tree

**DOI:** 10.3390/plants12213664

**Published:** 2023-10-24

**Authors:** John Paul Bradican, Salvatore Tomasello, Francesco Boscutti, Kevin Karbstein, Elvira Hörandl

**Affiliations:** 1 Department of Systematics, Biodiversity and Evolution of Plants (with Herbarium), Albrecht-von-Haller Institute for Plant Sciences, University of Göttingen, Untere Karspüle 2, 37073 Göttingen, Germany; 2 Georg-August University School of Sciences (GAUSS), University of Göttingen, Wilhelmsplatz 1, 37073 Göttingen, Germany; 3 Department of Agricultural, Food, Environmental and Animal Sciences, University of Udine, Via delle Scienze 91, 33100 Udine, Italy; 4 Department of Biogeochemical Integration, Max Planck Institute for Biogeochemistry, Hans Knöll Strasse 10, 07743 Jena, Germany

**Keywords:** species complex, hybridization, apomixis, polyploidy, Mediterranean

## Abstract

The taxonomic status of many Southern European taxa of the *Ranunculus auricomus* complex remains uncertain despite this region’s proximity to the native ranges of the sexual progenitor species of the complex. We investigated whether additional sexual progenitor species are present in the Mediterranean region. Utilizing target enrichment of 736 single-copy nuclear gene regions and flow cytometry, we analyzed phylogenomic relationships, the ploidy level, and the reproductive mode in representatives of 16 populations in Southern Europe, with additional sequence data from herbarium collections. Additionally, phased sequence assemblies from suspected nothotaxa were mapped to previously described sexual progenitor species in order to determine hybrid ancestry. We found the majority of Mediterranean taxa to be tetraploid, with hybrid populations propagating primarily via apomixis. Phylogenomic analysis revealed that except for the progenitor species, the Mediterranean taxa are often polyphyletic. Most apomictic taxa showed evidence of mixed heritage from progenitor species, with certain progenitor genotypes having mapped more to the populations from adjacent geographical regions. Geographical trends were found in phylogenetic distance, roughly following an east-to-west longitudinal demarcation of the complex, with apomicts extending to the southern margins. Additionally, we observed post-hybridization divergence between the western and eastern populations of nothotaxa in Southern Europe. Our results support a classification of apomictic populations as nothotaxa, as previously suggested for Central Europe.

## 1. Introduction

The shift brought about by the end of the 20th century from morphology-based taxonomic systems towards classification informed by genetics has resulted in a myriad of new insights into the evolution of plants [1,2,3]. Recently, increasing sophistication and scale in the collection and interpretation of sequence and other molecular data has allowed for increased resolution in the delimitation of species and taxonomic groups on both shallow and deep timescales [4,5,6,7]. This has accompanied an intensification of research into species complexes, that is, groups of species and/or hybrids which share a common ancestry but with complex, reticulated interrelationships often exhibiting high morphological diversity, hybridization, and polyploidy [8,9,10,11,12,13].

The study of species complexes presents opportunities towards furthering our understanding of the process of speciation, particularly during the early development of reproductive barriers [14,15,16,17,18]. It has become apparent that whole-genome duplication (WGD) and the accompanied polyploidy has acted as a catalyst for speciation during the history of angiosperm evolution [3,19,20]. Contributing to the possible proactive effect of polyploidy on speciation are the associated effects including novel-trait acquisition (e.g., from neo-functionalization of gene copies), increased vigor and/or stress-tolerance, which may facilitate range and niche expansion, as well as the increased likelihood of reproductive incompatibility with diploid progenitors or relatives [21,22,23]. A polyploid state may stem from fertilization between unreduced gametes, spontaneous doubling from mitotic errors, or via hybridization [23]. Allopolyploidy (polyploidy forming as a result of hybridization) is observed in many species complexes, generating added or even novel genomic and phenotypic complexity via the chimeric expression of parental subgenomes [24].

Polyploidy in plants is often connected to apomixis, the propagation of plants via asexually formed seeds [25]. Apomixis is often facultative, and the combination of hybrid origins and residual sexuality results in a huge diversity of genetic lineages with slight morphological differences that are interconnected via reticulate relationships [13,26]. The classification of such apomictic lineages has been notoriously difficult, and different approaches exist to treat them as species, nothotaxa, subspecies, or leave them unnamed, depending on their evolutionary history [14]. Such complexes can cover large geographical distributions, and often the apomictic taxa occupy a far larger range than their sexual relatives [27,28]. The abundance and wide distribution of apomictic taxa demands the careful investigation of phylogenetic relationships and distribution ranges of asexual lineages and the separation from sexual progenitors, in order to produce practicable classifications [16].

The *Ranunculus auricomus* complex is a plant species complex exhibiting high morphological diversity, with many members being polyploid (predominantly allopolyploids, ranging from tetra- to octoploid) and propagating via apomixis [29,30,31,32,33,34,35]. *Ranunculus auricomus* agg. is distributed from Southern Europe north to Greenland and east across Northern Asia into the Seward Peninsula, Alaska [36,37,38,39]. Taxonomically, four diploid and one tetraploid sexual species are recognized (*R. cassubicifolius* W. Koch, *R. envalirensis* Grau, *R. flabellifolius* Heuff. ex Rchb., *R. marsicus* Guss. & Ten., and *R. notabilis* Hörandl & Gutermann, respectively), which are theorized to be the primary progenitors of the hundreds of morphotypes present throughout Europe [36,40,41,42,43,44]. Three sexual progenitor species (*R. cassubicifolius*, *R. flabellifolius*, and *R. envalirensis*) diverged from a widespread common ancestor between 830 and 580 ka, with *R. marsicus* and *R. notabilis* diversifying some 500,000 years after (300–100 ka) this initial speciation event [45]. Diversification of the progenitor lineages occurred via isolation within separate glacial refugial regions, whereby cycles of glacial contraction and expansion may have led to occasional contact and genetic exchange between the diverging lineages [45]. Following the end of the last glacial maximum and expansion of suitable habitats, hybrids of these progenitor species formed and expanded to newly available habitats [45]. Possibly due to the advantages gleaned from high intragenomic heterozygosity and asexual reproduction, hybrid genotypes spread to occupy a vast majority of the current range of the species complex, in contrast to the range contraction which occurred during the diversification of the common ancestor of the complex into progenitor lineages [45,46]. Further hybridization between allopolyploid lineages has also contributed to a highly interlaced, reticulate network of relatedness between hybrid populations [47]. Research into *R. auricomus* agg. populations in Central Europe and Southern Scandinavia has revealed certain geographic patterns in genetic similarity, resulting in three to five clusters of populations with similar contributions from progenitor genomes, roughly following longitudinal gradients [47].

Here, we investigate the phylogenetic and reproductive status of Southern European members of *R. auricomus* agg., with a special focus on presumed hybrid taxa present in Spain, Italy, and Greece (File S1). Several taxa have been described in the region, with only the five aforementioned ‘progenitors’ having been studied in depth for their reproductive mode and phylogenetic placement [29,40,45]. As geographical patterns would theoretically suggest the occurrence of further sexual progenitor species in Southern Europe, we want to assess the mode of reproduction of these taxa [46]. We seek to elucidate whether the taxa in the Mediterranean region represent distinct early diverging clades or instead represent hybrid genetic clusters, as observed by Karbstein et al. [47]. If the pattern observed in Europe (north of the Alps) extends to the Mediterranean, do populations in Southern Europe descend primarily from the five known progenitor species, or do they possess genotypes with contributions from additional, unknown progenitors? If the former is the case, it is possible that similar geographic trends in ancestry are present, which may parallel those present in Central Europe.

## 2. Results

### 2.1. Somatic Ploidy and Reproductive Mode

#### 2.1.1. Somatic Ploidy

**Figure 1:**
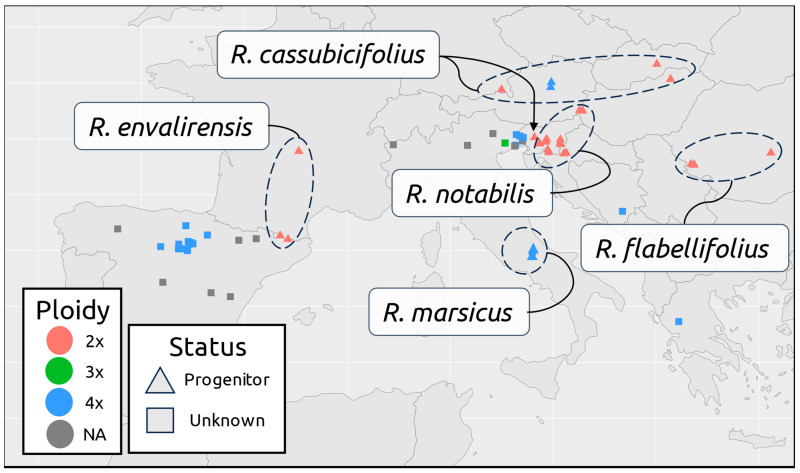
Locations of individuals used in the study. Locations of progenitor taxa are circled and labeled. Triangles = progenitor taxa, circles = presumed nothotaxa. Colors denote ploidy level. Full location data is listed in File S1.

Almost all nothotaxa sampled were found to be tetraploid, except for one Italian individual (‘*R.* ×*palaeoeuganeus* AD_1’), which is triploid (Figure 1). Ploidy data for individuals available only as herbarium specimens were taken from previous *R. auricomus* studies when available [48,49,50] (File S1).

#### 2.1.2. Reproductive Mode

Apomixis was found to be the dominant mode of reproduction for all newly measured populations (Table 1). Only one population (ES11094) was observed to reproduce sexually to some degree, although this is attributable to only two individuals (11094_5, 33% sexual seeds, 11094_4, 25% sexual seeds) (Table 1) (File S1).

### 2.2. Coalescent-Based Species Tree Estimation

After sequencing, an average of 1,291,196 reads per sample were recovered, ranging from 661,526 to 2,425,419. Following the processing and filtering steps implemented with the HybPhyloMaker pipeline, quality-checked reads averaged 1,233,679 per sample and ranged between 624,596 and 2,314,483 (see File S1). Supercontig recovery in HybPiper was successful for 70 individuals (see recovery statistics in File S2). Gene supercontigs were successfully aligned with MAFFT, and resolved gene trees were able to be calculated for 664 genes.

The phylogenetic analysis containing sexual progenitor species and outgroups shows high support, with multiple individuals of the same species from separate populations grouping together (Figure S1). *Ranunculus cassubicifolius* is resolved as the earliest diverging member of the complex. *Ranunculus flabellifolius* is a sister to a clade containing the remainder of the progenitor species (Figure S1).

**Figure 2:**
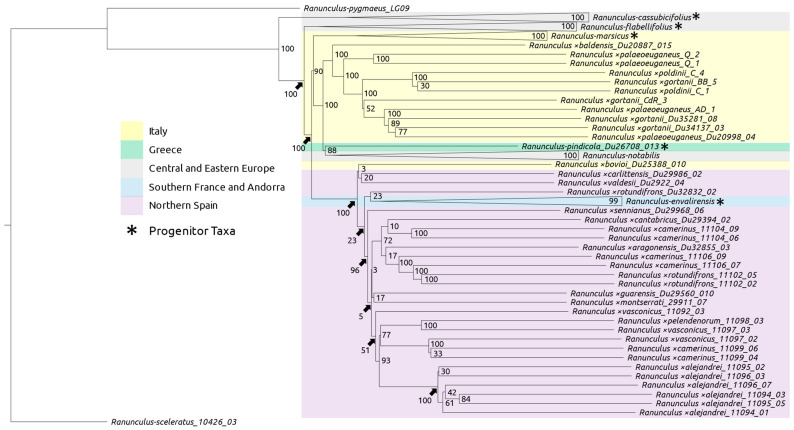
Species tree including all known sexual progenitor species, hybrid taxa, and outgroups (*R. sceleratus* and *R. pygmaeus*). The tree was calculated using ASTRAL-III with 100 multi-locus bootstrap repetitions (MLB). High support (≥90 MLB) is found only in basal nodes. Colors denote geographical origin; asterisks indicate sexual progenitor species.

Species tree estimation for all taxa resulted in lower support overall (Figure 2). High support is found for the branches containing progenitor species (except *Ranunculus envalirensis*), *Ranunculus pindicola*, and the basal nodes of the clade containing all Italian individuals) (Figure 2). The large, more distal clade containing all Spanish individuals and *Ranunculus envalirensis* shows low support for the basal nodes (Figure 2). Well-supported nodes largely diverge in correlation with the geographic location, except for Central European progenitor taxa (Figure 2). One clade contains Central European, Greek, Southeastern European, and Italian individuals, whereas the other includes French and Spanish individuals as well as the Northwestern Italian *R.* ×*bovioi* (Figure 2).

### 2.3. Phasing of Suspected Nothotaxa

**Figure 3:**
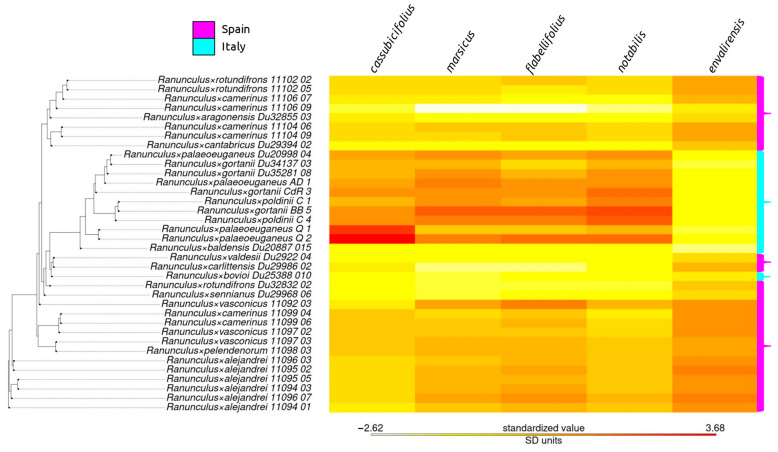
Heatmap of similarity between progenitor genotype reference individuals and suspected hybrid taxa. From left to right: *R. cassubicifolius*, *R. marsicus*, *R. flabellifolius*, *R. notabilis,* and *R. envalirensis*. Color bars on the right (Purple = Spain, Light Blue = Italy) indicate geographic origin. The species tree was calculated using ASTRAL-III as in Figure 2 with tips of non-nothotaxa and low branch length values pruned (see Methods). Here, only the tree topology is shown. Mapping performed in HybPhaser, normalized across reference genotypes.

HybPhaser detected similarities in most suspected hybrid individuals to the five progenitor species (Figure 3, detailed clade association values are listed in File S1). Low similarity was found in *R.* ×*baldensis*, *R.* ×*camerinus* 11106_09, *R.* ×*valdesii*, and *R. pindicola* (Figure 3). Similarly, the progenitor genotypes were associated broadly with the geographical location (Figure 3). High similarity to *R. envalirensis* was found only in Spanish populations/individuals, whereas high similarity to *R. notabilis* was found primarily in Italian populations/individuals (Figure 3). SNPs associated with *R. cassubicifolius*, *R. marsicus*, and *R. flabellifolius* were detected at a higher frequency in Italian individuals than in Spanish individuals, although certain Spanish nothotaxa also contained many SNPs from representatives of these species (e.g., *R.* ×*vasconicus* 11092_03; Figure 3).

**Figure 4:**
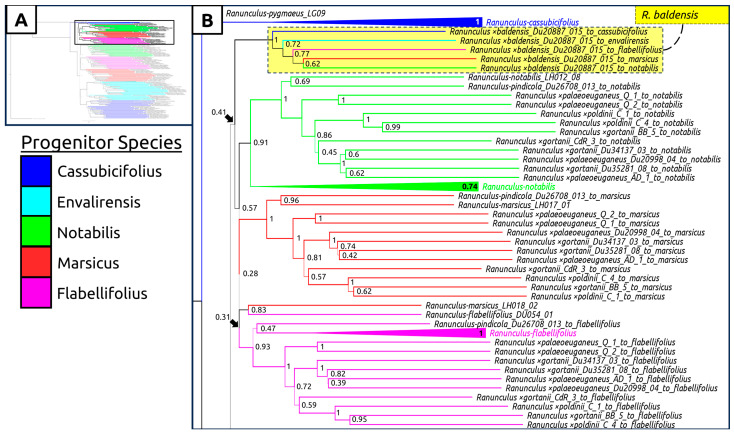
Multilabeled species tree created using phased sequences from suspected hybrid taxa. Legend is shown to the right of box (**A**). Color corresponds to the progenitor taxon to which the subgenomic contigs comprising the full-phased sequence were mapped to (legend left). (**A**) Full tree, showing region displayed in (**B**) (top, non-transparent rectangle). (**B**) Clade containing Italian subgenomic sequences mapping to *R. notabilis*, *R. marsicus*, and *R. flabellifolius* (in brackets). Additionally, a clade containing all subgenomic sequences from *R.* ×*baldensis* is shown (yellow). The tree was computed using ASTRAL-III with 100 multi-locus bootstrap repetitions. Line thickness and node values indicate multilocus bootstrap support.

**Figure 5:**
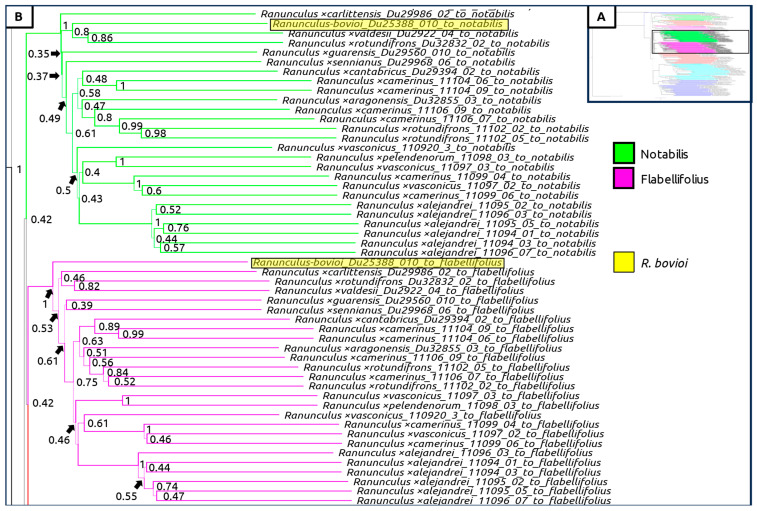
Multilabeled species tree created using phased sequences from suspected hybrid taxa. Legend is shown to the bottom of box (**A**). Color corresponds to the progenitor taxon to which the subgenomic contigs comprising the full-phased sequence were mapped to. (**A**) Full tree, showing region displayed in (**B**) (upper-middle, non-transparent rectangle). (**B**) Clades containing subgenomic sequences mapping to *R. notabilis* (green) and *R. flabellifolius* (pink), including Spanish taxa and western Italian *R. bovioi* which is highlighted. Line thickness and node values indicate multilocus bootstrap support. Computed using ASTRAL-III with 100 multi-locus bootstrap repetitions.

**Figure 6:**
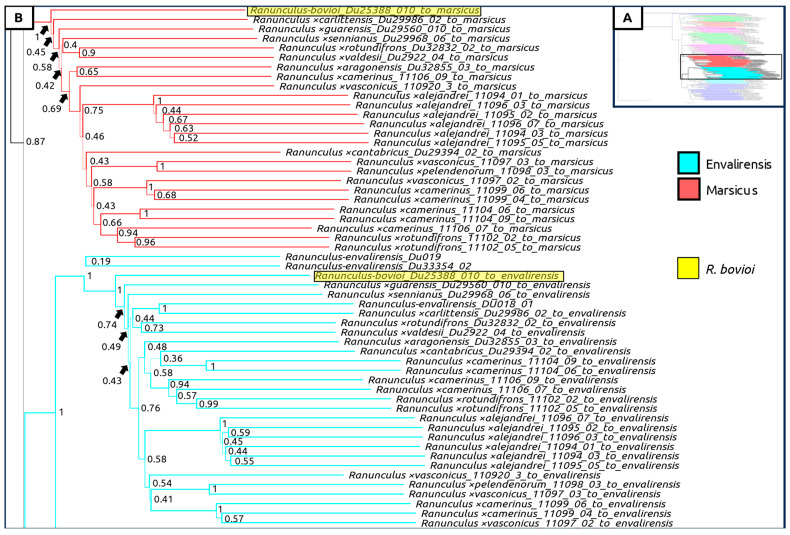
Multilabeled species tree created using phased sequences from suspected hybrid taxa. Legend is shown underneath the bottom of box (**A**). Color corresponds to the progenitor taxon to which the subgenomic contigs comprising the full-phased sequence were mapped to. (**A**) Full tree, showing region displayed in (**B**) (lower-middle, non-transparent rectangle). (**B**) Clades containing subgenomic sequences mapping to *R. envalirensis* (light blue) and *R. marsicus* (red), including Spanish taxa and western Italian *R. bovioi* which is highlighted. Line thickness and node values indicate multilocus bootstrap support. Computed using ASTRAL-III with 100 multi-locus bootstrap repetitions.

**Figure 7:**
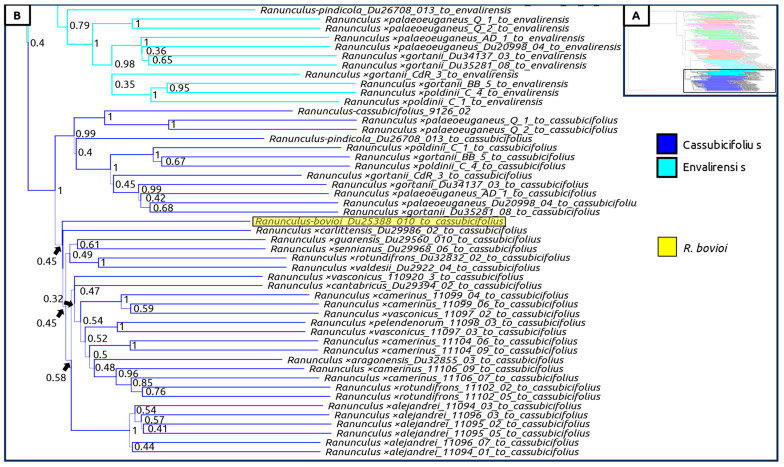
Multilabeled species tree created using phased sequences from suspected hybrid taxa. Legend is shown under the bottom of box (**A**). Color corresponds to the progenitor taxon to which the subgenomic contigs comprising the full-phased sequence were mapped to. (**A**) Full tree, showing region displayed in (**B**) (bottom, non-transparent rectangle). (**B**) Clade containing Italian subgenomic sequences mapping to *R. envalirensis* (light blue) and *R. cassubicifolius* (dark blue); Spanish taxa, and *R.* ×*bovioi* (yellow); a Western Italian individual is highlighted. Line thickness and node values indicate multilocus bootstrap support. Computed using ASTRAL-III with 100 multi-locus bootstrap repetitions.

A species tree generated with all sexual taxa as well as subgenomic contigs of suspected hybrid individuals was generated, showing again a clear geographic split between Western and Eastern populations (Figure 4, Figure 5, Figure 6 and Figure 7). Curiously, *Ranunculus* ×*baldensis* Du20887015 appears to be highly distinct, with all subgenomic contigs from this individual clustering together (Figure 4). Additionally, *Ranunculus* ×*bovioi* Du25388010, a Western Italian individual, consistently clusters with Spanish clades, as shown in the unphased species tree (Figure 5, Figure 6 and Figure 7). *Ranunculus pindicola* Du26708013, from Northern Greece, clusters with Italian clades, as shown in the unphased species tree (Figure 2 and Figure 4). Italian sequence regions which are mapped to the progenitor species *R. envalirensis* and *R. cassubicifolius* were grouped into a large clade containing *R.* ×*bovioi* Du25388010 and the Spanish taxa (Figure 6 and Figure 7).

## 3. Discussion

The classification of taxa within many species complexes has proved challenging to taxonomists for some time [40,47]. In *R. auricomus* agg., this is further complicated by facultative apomictic propagation, resulting in some reticulation of the genome pools via hybridization between allopolyploid populations and backcrossing to sexual species, though facultative sexual reproduction appears to be uncommon in European polyploids [45,46,50]. The hybrid origin of polyploid apomictic *R. auricomus* agg. populations also contributed greatly to the chimeric nature of their genomes via hybrid segregation which likely preceded whole genome duplication [47]. We find polyploidy and near-obligate apomictic propagation to be the norm in our sampling of Southern European populations (Figure 1, Table 1). Additionally, our data indicate a possible allopolyploid origin for many apomictic Italian and Spanish *R. auricomus* complex members (Figure 3). However, in some cases, for example, regarding *R.* ×-*baldensis*, evidence for hybrid ancestry is lacking in the similarity analysis on single individuals (Figure 3). The taxon might represent an allopolyploid with one unknown/extinct ancestor.

A clear demarcation between sexual and asexual reproduction is observed when comparing the reproductive modes of *R. auricomus* agg. progenitor species (which only rarely reproduce asexually) and other members of the complex found in Italy and Spain (Table 1, File S1) [40,46]. The degree of sexual reproduction is very low in nothotaxa, and in our dataset is isolated to a single individual (Table 1). However, it is important to consider that the reproductive data gathered here represent a ‘snapshot’ into the mode of reproduction of the Italian and Spanish populations during the years 2021 and 2022, respectively (File S1). It is possible that individuals vary from year to year in the degree to which they reproduce asexually, possibly according to the environmental conditions as observed in climate chamber experiments with varying light intensity and temperature [51,52]. Interestingly, the Spanish population including the two sexual reproducing individuals is located on an old pasture, fully exposed to the sun, whereas all other populations in Spain grow in deep shade in beech and oak forests. This corroborates the previous results that sexuality in *R. auricomus* is correlated to higher light intensity [46]. For future studies, it would be prudent to utilize seed collections from multiple years, and if variation is present, to take environmental factors into consideration. Additionally, though previous investigations into *R.* ×*baldensis* established high pollen quality, no FCSS analyses have been conducted from the material gathered from this taxon [53]. Owing to the dissimilarity between *R.* ×*baldensis* and the suspected nothotaxa examined here, further investigation into the reproductive mode of this taxon is warranted (Figure 3 and Figure 4).

Species relationships between progenitor taxa are reproduced here, largely conforming to previous estimates using different methodologies (Figure S1) [40,45]. Although some individuals show low similarity to progenitor species (most notably *R.* ×-*baldensis* Du20887015, *R.* ×-*valdesii* Du292204, *R. pindicola* Du26708013, and *R.* ×-*sennenianus* Du2996806), it is difficult to make concrete conclusions on ancestry given our data (Figure 4, Figure 5, Figure 6 and Figure 7). For example, *R. pindicola* is remarkably isolated compared to the rest of the taxa examined here and may therefore represent a divergent, earlier branching lineage [50]. Morphologically, *R. pindicola* resembles *R. notabilis* in having the same type of leaf cycle, but with less divided and more pedate spring leaves and glabrous receptacles, suggesting a mosaic-like character combination as typical for apomictic hybrids rather than for an autopolyploid [50,54]. The population is tetraploid and has very low pollen quality as typical for apomicts [50]. However, this uncertainty of relationships is possibly a result of under-sampling in the Balkans (personal communication P. Schönswetter). For instance, the recent finding of an (unnamed) apomictic population in Bosnia-Herzegovina (Figure 1) is a new record of the *R. auricomus* agg. for this country [44]. Glacial refugia in Southeastern Europe during the Pleistocene were likely numerous with dynamic borders, entailing complex effects on future genetic diversity of allopatrically isolated lineages and descendants [55,56]. It has been proposed that some progenitor species of the *R. auricomus* complex may no longer be extant as sexually reproducing populations, instead being detectable only as subgenomic regions in modern polyploid populations [47]. Certain distinctive taxa examined here, such as *R.* ×*baldensis*, may therefore represent the descendants of a currently undiscovered or extinct progenitor species.

Species tree estimation using coalescent-based methods showed poor resolution for the majority of nothotaxa (Figure 2). Particularly within the clade containing *R. envalirensis* and Spanish taxa, multi-locus bootstrap support is often extremely low (Figure 2). This is likely due to a recent history of hybridization in allopolyploid lineages and low divergence between lineages [40,47]. The chimeric nature of allopolyploid genomes challenges traditional species–concept notions [16]. We find significant similarities to progenitor reference genotypes in most polyploid taxa (Figure 3), supporting previous interpretations of three European ‘clusters’ of reticulated allopolyploid populations which may be extended to Southern European *R. auricomus* agg. [47].

We find groups of populations and individuals with high similarity to one another, likely due to the young age of such lineages, and partly attributable to asexual propagation (Figure 2) [57]. Additionally, geographic trends are observed according to similarity to progenitor species reference genotypes as well as phylogenetic distance (Figure 3 and Figure 4). Italian populations, apart from *R.* ×*bovioi*, are observed to typically have low similarity to *R. envalirensis* with higher similarity to the other four progenitor species (Figure 3). Conversely, most Spanish individuals show affinity to the *R. envalirensis* reference genotype, with varying contributions from *R. cassubicifolius*, *R. marsicus,* and *R. flabellifolius*, and lower similarity to *R. notabilis* (Figure 3). It is possible that this is due to geographical proximity, as *R. envalirensis* is the western-most progenitor species (located in Southern and Western France) with the other four progenitor species located east of the 14th degree longitude into Southern Italy, Central Europe and Eastern Europe (Figure 1). *R.* ×*bovioi* represents an interesting exception to this trend, with relatively balanced admixture from progenitor lineages, possibly due to contact between allopolyploids originating from distinct contact zones (Figure 3). As glaciers receded at the end of the last glacial maximum and range expansion of allopolyploid *R. auricomus* agg. began, contact zones may have been more likely to form towards the east of the complex’s range [45]. In contrast, in Southwestern Europe hybridization between *R. envalirensis* and other sexual progenitor species would have been less likely, barring the presence of now extinct progenitor species. Nonetheless, some contributions from eastern progenitor species are detected in Spanish populations suggesting either the presence of a contact zone along the Ligurian coast, or possibly dispersal of eastern populations to the west. However, hybrid origin could also predate the last glacial maximum, and the sexual progenitors could have had larger distributions and contact zones in earlier warmer periods of the Pleistocene, thus leaving genetic signatures in Spanish populations [47]. Expanded contact zones and/or dispersal of allopolyploids with admixture from *R. envalirensis* and *R. cassubicifolius* into Italy and the Balkans may also explain the detection of ancestry from these lineages in some Italian and Balkanic individuals (Figure 3).

Our results confirm a geographical parthenogenesis scenario in *R. auricomus* but refine the pattern to a more central distribution of sexual progenitors and expansion of the apomicts not only towards the North, but also to the southern margins of the distribution range [28,46,58,59]. This pattern is in line with a superior colonization ability of apomictic plants due to uniparental reproduction [28]. The higher light intensity in more Southern regions could stimulate sexuality, but in the Iberian Peninsula this is probably buffered by very shaded habitats in forest floors, mostly situated in beech forests ranging from 1000 and 1400 m altitude [51]. More detailed studies on populations in the southern range are wanted to understand ecological factors.

## 4. Materials and Methods

### 4.1. Sampling

Material for further analyses was taken from *R. auricomus* agg. both in situ, and ex-situ (individuals collected as both herbarium and living specimens). GPS coordinates, altitude and identifying information for all individuals collected as living specimens are available in File S1. Living specimens were collected and stored at the Old Botanical Garden at the University of Göttingen, and material gathered from these individuals included: (1) silica-dried leaf material for target enrichment sequencing and somatic ploidy determination, and (2) ripe seeds for flow cytometric seed screening and determination of reproductive mode. Individuals collected as herbarium specimens were utilized only in target enrichment sequencing, where leaf tissue was gathered from stem leaves of the dried material. GPS coordinates were not available for all herbarium specimens used, and approximate coordinates gathered from the description of the collector were used (see File S1). The data pertaining to southern European *R. auricomus* agg. species were collected from the literature [45,48,49,50,53].

In the case of populations where herbarium specimens were available, one representative of a population was used for target enrichment and phylogenetic analyses. For populations which were collected in situ or as living specimens, material from multiple individuals was collected. For these populations, two individuals were sequenced via target enrichment, and between three and five were analyzed for somatic and reproductive ploidy levels using flow cytometry (File S1).

### 4.2. Somatic Ploidy and Reproductive Mode Determination

The ploidy levels of leaf, embryo and endosperm tissue were estimated using flow cytometry. For somatic ploidy, silica-dried leaf tissue (~0.5–1 cm^2^) was collected and pulverized into small fragments by placing the leaf tissue in a 2 mL Eppendorf tube with a 4 mm diameter steel ball, subsequently placed in a Tissue Lyzer II (Qiagen, Hilden, Germany) and run for 10–15 s at a 30 Hz frequency [46]. Somatic ploidy measurements were carried out for all individuals for which silica-dried leaf material was available, collected from garden individuals and/or in situ populations (Table 1). Measurements were made using a CyFlow Ploidy Analyzer (Sysmex, Nordstedt, Germany) in conjunction with CUBE16 v.1.6 software (Sysmex, Nordstedt, Germany) [52]. For all individuals from Spanish populations, deionized H_2_O was used in place of sheath fluid. The measured median size of intact nuclei (within a target region, or ‘peak’) was compared to the nuclei size of a diploid *R. cassubicifolius* standard, and ploidy was inferred based on this standard metric.

In order to determine whether individual seeds were produced via sexual reproduction or apomixis, single-seed flow cytometric screening was conducted [46,60]. Seeds were collected in situ during the years of 2021 and 2022 and stored at ~2–3 °C. For extracting the nuclei from the seed tissue, a previously described protocol was followed [40]. The data were gathered as above using a CyFlow Ploidy Analyzer (Sysmex, Nordstedt, Germany) with CUBE16 v.1.6 software (Sysmex, Nordstedt, Germany) installed. In order to determine the reproductive mode, the median nuclei size of intact embryo and endosperm nuclei were compared by generating a peak index (PI) metric, where the inferred ploidy of the endosperm nuclei (determined via reference to a standard, see above) is divided by the inferred ploidy of embryonic nuclei. In the pseudogamous apomictic pathway observed in *R. auricomus* agg., a seed with a PI between 1.7 and 2 is determined to be produced sexually, whereas peak indices above 2 indicate that the seed was produced via apomixis [40]. In measurements where endosperm ploidy was measurable and two times that of the somatic ploidy of the plant, but the embryo ploidy was not determined, we used the somatic ploidy in lieu of the embryo in order to determine the PI.

### 4.3. DNA Extraction and Target Enrichment

DNA was extracted from ~1.5 cm^2^-sized probes of silica-dried and herbarium leaf material using the Qiagen Dneasy Plant Mini Kit (Qiagen, Hilden, Germany). A modified protocol for use in the genus was followed (File S2).

A target enrichment workflow detailed in (cite) was followed, utilizing a custom bait set consisting of 17,988 probes targeting 736 low-copy nuclear genomic regions [45]. The concentration of amplified target regions differed between rounds of sequencing according to varying pooling schemes, and the target enrichment protocols used are detailed in File S2. Sequencing was performed on an Illumina MiSeq System (Illumina, San Diego, CA, USA) for two paired-end sequencing runs.

DNA extraction was successful for 36 individuals from 27 populations and the target regions of the nuclear genome were successfully amplified for 36 individuals from 27 populations (File S1).

We also utilized a previous dataset for the inclusion of the sexual progenitor species as well as two outgroup taxa (*Ranunculus pygmaeus* and *Ranunculus sceleratus*) [45].

### 4.4. Phylogenomic Analyses

The raw sequence data were first quality-checked and trimmed using the first two steps of the HybPhyloMaker pipeline which trims adaptor sequences and low-quality reads [61]. Afterwards, these reads were passed on to the HybPiper pipeline [62].

A target file was first generated from the probe sequences for use with HybPiper (File S2). HybPiper was run using the assemble, intronerate, and paralog retriever functions (see File S2 for details), where bwa was used for mapping [63,64]. Gene supercontigs with introns, produced via HybPiper, were aligned using MAFFT v7.305b [65]. Gene trees were calculated from MAFFT gene alignments using IQTREE multicore version 1.6.12. IQTREE was run using ModelFinder Plus, 1000 UFBoot replicates (in conjunction with the -bnni function in order to avoid severe model violations) [66,67,68]. A multi-species coalescent (MSC) consistent estimation of species trees was then calculated using ASTRAL-III with 100 multi-locus bootstrap replicates [69]. Although taxa within the *R. auricomus* complex predominantly evolve in a reticulate manner violating assumptions of many model-based phylogenetic approaches, tree conflicts can give first insights into the reticulate evolution of newly examined taxa [47]. This analysis was performed for a sample set containing both (1) progenitor species, *Ranunculus pindicola*, and outgroups (*Ranunculus pygmaeus* and *Ranunculus sceleratus*), and (2) all individuals, including outgroups.

In order to estimate the degree to which progenitor species’ subgenomic elements were present in polyploid individuals, the clade association functions of the HybPhaser pipeline was used [70,71]. HybPhaser configurations are listed in File S2. Individuals selected as representatives for progenitor species were selected according to the following criteria: (1) high coverage and on-target reads from HybPiper output compared to other individuals of the same species, and (2) low allelic divergence and locus heterozygosity. Due to incompatibilities between the ASTRAL-III branch length support values and the phylo.heatmap function in phytools, not all polyploid taxa are shown in Figure 3. All clade association values are listed in File S2. Phased sequences generated via HybPhaser were utilized in another run of HybPiper, run using the same settings listed above and in File S2. Recovered supercontigs were aligned, and gene trees were calculated as above. Species tree determination for the phased polyploid, unphased polyploid, and diploid sequences was performed using ASTRAL-III as detailed above and in File S2.

Analyses were visualized in FigTree v1.4.4 and R version 4.2.2 using the phytools package [72].

## 5. Conclusions

Members of the *Ranunculus auricomus* species complex are present in Southern Europe, where not one of the five recognized early diverging progenitor lineages, they reproduce primarily asexually, and they are most often tetraploid. Evidence for a hybrid origin of these polyploids is present in many of the individuals examined here; many of whom belong to taxa which we find to be likely polyphyletic. Some divergence along the geographic lines is observed, although these are likely young lineages and this may also represent similar hybrid origins.

## Figures and Tables

**Table 1:** Overview of reproductive modes (apomictic or sexual) from Bosnian, Italian, and Spanish *R. auricomus* agg. populations. Seeds were collected in situ and analyzed using single-seed flow cytometric screening (measurements and locations given in File S1). Percentages were calculated from means over all seeds per population.

Population ID	Country	Number of Individuals	Number of Seeds	Percent Apomictic	Percent Sexual	Individuals Sexual
17842	BA	1	5	100	0	0
Q	IT	3	28	100	0	0
AD	IT	2	19	100	0	0
BB	IT	3	29	100	0	0
BosBo	IT	3	26	100	0	0
CdR	IT	3	27	100	0	0
C	IT	3	27	100	0	0
11092	ES	5	25	100	0	0
11094	ES	6	24	91.30	8.70	2:11094_5, 33% Sexual; 11094_4, 25% Sexual
11096	ES	2	10	100	0	0
11097	ES	2	11	100	0	0
11098	ES	2	19	100	0	0
11099	ES	3	25	100	0	0
11102	ES	3	34	100	0	0
11104	ES	4	32	100	0	0
11105	ES	1	10	100	0	0
11106	ES	3	25	100	0	0

## Data Availability

New sequence data generated for this research is available on the Göttingen Research Online database, accessible via: https://doi.org/10.25625/MNZ9O8.

## Supplementary Materials

The following supporting information can be downloaded at: https://www.mdpi.com/article/10.3390/plants12213664/s1, Figure S1: Phylogeny of Progenitor Species; File S1: List of Individuals and Flow Cytometry Data; File S2: Molecular Data Appendix and Details on Phylogenomic Methods Used.
