# Supplementary material for: Phylogenomics of Southern European Taxa in the Ranunculus auricomus Species Complex: The Apple Doesn’t Fall Far from the Tree
Source: Plants (Basel). 2023 Oct 24;12(21):3664. doi: 10.3390/plants12213664 (PMC10650656; doi:10.3390/plants12213664)
Supplement: Supplementary file 1 [file plants-12-03664-s001.zip › AppendixS2_2023_Plants_revised.docx]

**Library Preparation**

Target enrichment libraries were produced largely following the protocol detailed in Tomasello et al. 2020 (cite). We utilized the same custom bait-set detailed by Tomasello et al. 2020 (cite). DNA extraction was performed utilizing a modified protocol of the Qiagen DNeasy Plant Mini Kit® (Qiagen, Hilden, Germany), with a total initial incubation of the sample material in lysis buffer for one hour. Fragment size and quality of total DNA extracts were determined via gel electrophoresis, using 1.5% agarose gels, and Roti®-Load DNAstain 3 (Carl Roth, Karlsruhe, Germany) for visualization of genetic material. The concentration of DNA extracts were determined using a Qubit® fluorometer in conjunction with the Qubit® dsDNA HS Assay Kit (ThermoFisher Scientific, Waltham, USA). DNA extraction was successful for 36 individuals from 27 populations. Target regions were successfully amplified for 36 individuals from 27 populations.

For target enrichment, the “NEBNext Ultra II FS DNA Library Prep Kit for Illumina®” (E7805) (New England BioLabs, Ipswich, USA) was used. As enzymatic shearing is utilized in this protocol, and the fragmentation of our DNA extracts varied (according to age, quality of storage, etc.) the incubation for this step consequently varied accordingly, ranging from 1-12 minutes.

**Processing of reads, contig assembly, alignments, gene and species tree calculation**

Raw sequence data was first quality-checked and trimmed using the first two steps of the HybPhyloMaker pipeline which removes adaptor sequences and performs quality filtering, full statistics from quality trimmed sequences are listed in AppendixS2 [61]. Afterwards, these reads were passed on to the HybPiper pipeline [62]. Note that in Recovery and trimming statistics listed below and in AppendixS2: due to formatting requirements in HybPhyloMaker, the individual code follows directly after the population code without an underscore, sometimes preceded by an ‘S’.

A target file was first generated from probe sequences for use with HybPiper, according to the guide for target file preparation (<https://github.com/mossmatters/HybPiper/wiki#12-target-file>) [62]. HybPiper was run using the assemble, intronerate, and paralog retriever functions, utilizing GNU Parallel [63]. Gene supercontigs, including introns when possible, produced by HybPiper were aligned using MAFFT v7.305b [65]. Gene trees were calculated from MAFFT gene alignments using IQTREE [68]. IQTREE was run with the following parameters: 1000 UFBoot replicates, ModelFinder plus (individually performed for each gene contig), and the -bnni function in order to alleviate model violations [65-67]. A multi-species coalescent (MSC) consistent estimation of species trees was then calculated using ASTRAL-III with 100 multi-locus bootstrap replicates [69].

HybPhaser was utilized, incorporating output from HybPiper (see configuration used below) [70]. Clade association analysis was performed in order to track the degree of similarity between suspected hybrid taxa and progenitor genotypes, shown in Figure 4 (heatmap). The following representatives were chosen to represent progenitor taxa, based on low allelic divergence, high coverage, and low locus heterozygosity:

Ranunculus-cassubicifolius_9126_02 - “cassubicifolius clade”

Ranunculus-envalirensis_DU018_01 - “envalirensis clade”

Ranunculus-flabellifolius_LH25_01 - “flabellifolius clade”

Ranunculus-marsicus_LH017_01 - “marsicus clade”

Ranunculus-notabilis_LH01208 - “notabilis clade”

HybPhaser includes the option to pull contiguous sequences mapping to clade representatives via BBSplit [71]. Sequences obtained in this manner were then passed through the same HybPiper – MAFFT – IQTREE – ASTRAL-III pipeline detailed above for visualization.

**HybPiper Recovery 1 (Species only, Figure S1)**

Name NumReads ReadsMapped PctOnTarget GenesMapped GenesWithContigs GenesWithSeqs GenesAt25pct GenesAt50pct GenesAt75pct GenesAt150pct ParalogWarningsLong ParalogWarningsDepth GenesWithoutStitchedContigs GenesWithStitchedContigs GenesWithStitchedContigsSkipped GenesWithChimeraWarning

Ranunculus-cassubicifolius_9126S02 1488270 822866 55.3 683 635 633 576 435 264 0 6 7 337 296 0 0

Ranunculus-cassubicifolius_DU013S01 1303340 960900 73.7 678 663 661 651 631 618 0 33 84 214 447 0 0

Ranunculus-cassubicifolius_LH006S17 1211924 821255 67.8 680 663 661 647 632 619 0 33 69 202 459 0 0

Ranunculus-cassubicifolius_LH016S14 1856820 1292641 69.6 685 664 662 649 632 617 0 30 71 210 452 0 0

Ranunculus-cassubicifolius_LH08S07 1255598 850809 67.8 684 663 663 646 627 614 1 47 109 192 471 0 0

Ranunculus-cassubicifolius_LH09S03 1001666 682904 68.2 682 661 661 646 628 608 0 32 81 196 465 0 0

Ranunculus-cassubicifolius_LH40S04 993306 649379 65.4 684 661 660 647 627 614 1 30 64 187 473 0 0

Ranunculus-envalirensis_DU018S01 803072 553514 68.9 678 660 660 647 629 612 0 31 66 214 446 0 0

Ranunculus-envalirensis_Du019 951898 515528 54.2 686 663 663 647 629 611 0 32 55 198 465 0 0

Ranunculus-envalirensis_Du33354S02 1277390 777754 60.9 679 663 663 647 628 596 0 27 55 190 473 0 0

Ranunculus-flabellifolius_DU021S01 1321328 935039 70.8 675 663 662 647 630 614 0 34 71 193 469 0 0

Ranunculus-flabellifolius_DU054S01 942820 693809 73.6 680 662 662 651 632 617 0 40 81 221 441 0 0

Ranunculus-flabellifolius_LH25S01 897624 563184 62.7 675 661 661 647 631 607 0 27 50 198 463 0 0

Ranunculus-marsicus_LH017S01 906332 672415 74.2 677 659 659 647 631 610 0 43 92 222 437 0 0

Ranunculus-marsicus_LH018S02 1212884 847517 69.9 677 663 661 648 631 614 0 45 106 210 451 0 0

Ranunculus-notabilis_10137S03 1172086 919057 78.4 679 662 662 648 632 616 0 37 74 208 454 0 0

Ranunculus-notabilis_2 991036 263526 26.6 688 652 652 638 614 569 1 27 46 192 460 0 0

Ranunculus-notabilis_DU003S01 762410 607301 79.7 677 660 660 648 630 607 1 37 78 218 442 0 0

Ranunculus-notabilis_DU049S01 1140030 898036 78.8 682 665 663 650 630 616 1 38 86 213 450 0 0

Ranunculus-notabilis_Du35351S15 1547866 913260 59.0 680 662 661 645 625 594 0 24 53 187 474 0 0

Ranunculus-notabilis_Hoe5615 1143234 730324 63.9 684 661 660 643 615 563 1 21 42 207 453 0 0

Ranunculus-notabilis_LH010S07 734346 549065 74.8 677 658 658 645 629 611 1 39 76 212 446 0 0

Ranunculus-notabilis_LH011S01 1131922 839111 74.1 680 664 662 647 630 617 0 36 78 212 450 0 0

Ranunculus-notabilis_LH011S14 849882 637957 75.1 677 664 662 648 629 615 1 38 75 225 437 0 0

Ranunculus-notabilis_LH012S08 1048068 852072 81.3 677 662 662 651 633 616 0 33 75 212 450 0 0

Ranunculus-notabilis_LH013S03 846348 685777 81.0 678 661 661 648 631 614 1 33 73 203 458 0 0

Ranunculus-notabilis_LH014S03 1170400 943075 80.6 678 662 662 647 631 617 2 32 71 206 456 0 0

Ranunculus-notabilis_LH015S10 1283046 1032560 80.5 680 665 663 649 631 615 1 33 75 202 461 0 0

Ranunculus-notabilis_LH028S02 1831010 1317240 71.9 685 663 662 648 624 597 0 24 65 182 480 0 0

Ranunculus-pindicola_Du26708013 2140168 1373069 64.2 691 662 662 646 628 595 1 34 79 197 465 0 0

Ranunculus-pygmaeus_LG09 1591966 1302472 81.8 679 667 663 647 631 612 0 30 45 208 455 0 0

Ranunculus-sceleratus_10426S3 1839446 1444819 78.5 683 665 662 645 630 601 0 131 337 207 455 0 0

**HybPiper Recovery 2 (All individuals unphased, Figures 2+3)**

Name NumReads ReadsMapped PctOnTarget GenesMapped GenesWithContigs GenesWithSeqs GenesAt25pct GenesAt50pct GenesAt75pct GenesAt150pct ParalogWarningsLong ParalogWarningsDepth GenesWithoutStitchedContigs GenesWithStitchedContigs GenesWithStitchedContigsSkipped GenesWithChimeraWarning

Ranunculus-alejandrei_1109401 1006842 917441 91.1 673 660 660 646 628 609 1 27 90 186 474 0 0

Ranunculus-alejandrei_1109403 989478 764749 77.3 675 659 659 646 629 607 1 28 75 194 465 0 0

Ranunculus-alejandrei_1109505 1214362 967554 79.7 675 662 661 647 630 610 0 32 91 194 467 0 0

Ranunculus-alejandrei_1109502 824514 620049 75.2 675 660 660 647 629 605 1 29 73 204 456 0 0

Ranunculus-alejandrei_1109607 877614 670862 76.4 678 660 660 647 629 605 1 27 75 191 469 0 0

Ranunculus-alejandrei_1109603 824204 633512 76.9 677 660 660 646 629 606 1 25 62 191 469 0 0

Ranunculus-aragonensis_Du3285503 1166416 716614 61.4 682 656 654 624 554 431 0 17 28 282 372 0 0

Ranunculus-baldensis_Du20887015 1090520 589037 54.0 682 647 644 584 456 319 0 14 17 347 297 0 0

Ranunculus-bovioi_Du25388010 1383794 773886 55.9 681 659 657 622 527 378 0 12 15 320 337 0 0

Ranunculus-camerinus_1109906 923348 682229 73.9 677 661 661 648 632 600 1 25 72 179 482 0 0

Ranunculus-camerinus_1109904 889064 784962 88.3 674 659 659 644 628 591 0 23 67 181 478 0 0

Ranunculus-camerinus_1110409 958842 839977 87.6 675 658 658 645 624 593 0 27 66 184 474 0 0

Ranunculus-camerinus_1110406 931846 822482 88.3 671 657 657 645 625 598 0 22 67 183 474 0 0

Ranunculus-camerinus_1110609 595352 419518 70.5 674 601 601 572 490 350 0 2 3 235 366 0 0

Ranunculus-camerinus_1110607 794892 591474 74.4 676 654 654 639 617 568 1 19 43 184 470 0 0

Ranunculus-cantabricus_Du2939402 1652628 980265 59.3 686 661 661 642 596 525 0 19 45 232 429 0 0

Ranunculus-carlittensis_Du2998602 1150042 702618 61.1 684 658 655 630 540 412 0 14 22 295 360 0 0

Ranunculus-cassubicifolius_9126S02 1488270 822866 55.3 683 635 633 577 436 272 0 6 7 339 294 0 0

Ranunculus-cassubicifolius_DU013S01 1303340 960900 73.7 678 663 661 651 631 619 0 33 84 214 447 0 0

Ranunculus-cassubicifolius_LH40S04 993306 649379 65.4 684 661 660 648 628 615 1 30 64 188 472 0 0

Ranunculus-cassubicifolius_LH006S17 1211924 821255 67.8 680 663 661 648 632 619 0 33 69 203 458 0 0

Ranunculus-cassubicifolius_LH016S14 1856820 1292641 69.6 685 664 662 650 632 617 0 30 71 211 451 0 0

Ranunculus-cassubicifolius_LH08S07 1255598 850809 67.8 684 663 663 647 627 614 1 47 109 193 470 0 0

Ranunculus-cassubicifolius_LH09S03 1001666 682904 68.2 682 661 661 647 628 609 0 32 81 197 464 0 0

Ranunculus-envalirensis_Du33354S02 1277390 777754 60.9 679 663 663 647 629 599 0 27 55 188 475 0 0

Ranunculus-envalirensis_Du019 951898 515528 54.2 686 663 663 647 629 611 0 32 55 197 466 0 0

Ranunculus-envalirensis_DU018S01 803072 553514 68.9 678 660 660 648 629 613 1 31 66 213 447 0 0

Ranunculus-flabellifolius_DU021S01 1321328 935039 70.8 675 663 662 647 629 615 0 34 71 193 469 0 0

Ranunculus-flabellifolius_DU054S01 942820 693809 73.6 680 662 662 652 632 617 0 40 81 220 442 0 0

Ranunculus-flabellifolius_LH25S01 897624 563184 62.7 675 661 661 648 630 608 0 27 50 197 464 0 0

Ranunculus-gortanii_CdR3 1004524 858192 85.4 674 658 658 645 625 597 0 29 75 187 471 0 0

Ranunculus-gortanii_BB5 1042308 922379 88.5 670 661 661 646 627 610 0 32 74 193 468 0 0

Ranunculus-gortanii_Du3413703 1508244 880418 58.4 685 659 658 641 597 527 0 20 48 214 444 0 0

Ranunculus-gortanii_Du3528108 1482950 772288 52.1 685 659 659 646 621 596 0 36 75 189 470 0 0

Ranunculus-guarensis_Du29560010 1365436 815214 59.7 686 661 660 635 586 489 0 22 47 244 416 0 0

Ranunculus-marsicus_LH017S01 906332 672415 74.2 677 659 659 648 631 612 0 43 92 222 437 0 0

Ranunculus-marsicus_LH018S02 1212884 847517 69.9 677 663 661 649 631 615 0 45 106 209 452 0 0

Ranunculus-montserrati_2991107 1252130 762159 60.9 681 657 656 635 585 504 0 22 39 238 418 0 0

Ranunculus-notabilis_10137S03 1172086 919057 78.4 679 662 662 649 632 616 0 37 74 208 454 0 0

Ranunculus-notabilis_Hoe5615 1143234 730324 63.9 684 661 660 644 616 563 1 21 42 207 453 0 0

Ranunculus-notabilis_LH028S02 1831010 1317240 71.9 685 663 662 649 625 598 0 24 65 182 480 0 0

Ranunculus-notabilis_LH014S03 1170400 943075 80.6 678 662 662 648 632 617 1 32 71 205 457 0 0

Ranunculus-notabilis_LH015S10 1283046 1032560 80.5 680 665 663 650 631 616 0 33 75 205 458 0 0

Ranunculus-notabilis_DU003S01 762410 607301 79.7 677 660 660 649 630 608 1 37 78 219 441 0 0

Ranunculus-notabilis_LH012S08 1048068 852072 81.3 677 662 662 651 633 616 0 33 75 213 449 0 0

Ranunculus-notabilis_2 991036 263526 26.6 688 652 652 639 615 572 1 27 46 192 460 0 0

Ranunculus-notabilis_Du35351S15 1547866 913260 59.0 680 662 661 646 626 596 0 24 53 187 474 0 0

Ranunculus-notabilis_LH010S07 734346 549065 74.8 677 658 658 646 629 612 1 39 76 211 447 0 0

Ranunculus-notabilis_LH011S01 1131922 839111 74.1 680 664 662 647 630 617 0 36 78 211 451 0 0

Ranunculus-notabilis_LH011S14 849882 637957 75.1 677 664 662 649 629 615 1 38 75 224 438 0 0

Ranunculus-notabilis_DU049S01 1140030 898036 78.8 682 665 663 650 630 616 1 38 86 212 451 0 0

Ranunculus-notabilis_LH013S03 846348 685777 81.0 678 661 661 649 631 614 1 33 73 202 459 0 0

Ranunculus-palaeoeuganeus_Q2 1090402 894743 82.1 676 660 660 647 628 607 2 31 84 193 467 0 0

Ranunculus-palaeoeuganeus_AD1 860796 612987 71.2 674 652 652 639 620 579 0 28 59 186 466 0 0

Ranunculus-palaeoeuganeus_Q1 924218 650467 70.4 677 653 653 634 595 529 0 22 40 226 427 0 0

Ranunculus-palaeoeuganeus_Du2099804 1339500 678351 50.6 694 659 658 643 609 563 1 22 55 193 465 0 0

Ranunculus-pelendenorum_1109803 776298 555702 71.6 675 657 657 645 626 596 0 27 62 187 470 0 0

Ranunculus-pindicola_Du26708013 2140168 1373069 64.2 691 662 662 647 627 597 1 34 79 198 464 0 0

Ranunculus-poldinii_CB4 1056478 944602 89.4 672 658 658 645 627 600 1 29 75 182 476 0 0

Ranunculus-poldinii_C1 977756 700082 71.6 675 658 658 646 616 586 0 24 63 197 461 0 0

Ranunculus-rotundifrons_1110202 981596 865379 88.2 671 657 657 643 623 594 0 25 67 187 470 0 0

Ranunculus-rotundifrons_1110205 763864 666688 87.3 670 657 657 645 622 587 0 22 51 185 472 0 0

Ranunculus-rotundifrons_Du3283202 1662916 1064396 64.0 681 657 656 637 583 470 0 17 32 260 396 0 0

Ranunculus-sennianus_Du2996806 1322572 863852 65.3 682 658 656 632 579 456 2 18 25 269 387 0 0

Ranunculus-valdesii_Du292204 1632930 993487 60.8 687 661 659 631 578 483 0 22 38 249 410 0 0

Ranunculus-varsonicus_1109702 1161170 863057 74.3 680 661 661 646 631 610 1 29 76 190 471 0 0

Ranunculus-varsonicus_1109703 972210 729702 75.1 677 659 659 647 627 601 0 30 69 191 468 0 0

Ranunculus-vasconicus_1109203 1374356 1229348 89.4 675 661 661 648 631 616 0 31 88 192 469 0 0

Ranunculus-pygmaeus_LG09 1591966 1302472 81.8 679 667 663 648 631 612 0 30 45 210 453 0 0

Ranunculus-sceleratus_10426S3 1839446 1444819 78.5 683 665 662 645 630 601 0 131 337 207 455 0 0

**HybPiper Recovery 3 (All suspected hybrids phased + sexual species, Figures 4+5)**

Name NumReads ReadsMapped PctOnTarget GenesMapped GenesWithContigs GenesWithSeqs GenesAt25pct GenesAt50pct GenesAt75pct GenesAt150pct ParalogWarningsLong ParalogWarningsDepth GenesWithoutStitchedContigs GenesWithStitchedContigs GenesWithStitchedContigsSkipped GenesWithChimeraWarning

Ranunculus-alejandrei_1109401_to_cassubicifolius 306355 304051 99.2 628 474 474 409 217 100 0 4 4 336 138 0 0

Ranunculus-alejandrei_1109401_to_envalirensis 515469 509311 98.8 659 634 634 619 557 432 0 7 9 199 435 0 0

Ranunculus-alejandrei_1109401_to_flabellifolius 433138 428691 99.0 656 595 595 560 443 262 0 6 10 257 338 0 0

Ranunculus-alejandrei_1109401_to_marsicus 481446 476180 98.9 658 624 624 594 508 341 0 5 11 277 347 0 0

Ranunculus-alejandrei_1109401_to_notabilis 432373 427442 98.9 659 612 611 584 449 288 0 5 7 262 349 0 0

Ranunculus-alejandrei_1109403_to_cassubicifolius 251783 249854 99.2 628 475 475 397 210 85 0 1 2 356 119 0 0

Ranunculus-alejandrei_1109403_to_envalirensis 427938 422630 98.8 659 637 637 617 543 412 0 6 8 222 415 0 0

Ranunculus-alejandrei_1109403_to_flabellifolius 359256 355350 98.9 656 585 585 544 424 247 0 4 7 249 336 0 0

Ranunculus-alejandrei_1109403_to_marsicus 400795 396106 98.8 658 618 618 594 494 326 0 6 12 259 359 0 0

Ranunculus-alejandrei_1109403_to_notabilis 357946 353563 98.8 659 608 608 572 455 280 0 4 6 262 346 0 0

Ranunculus-alejandrei_1109502_to_cassubicifolius 201816 200493 99.3 629 450 450 377 199 81 0 3 3 330 120 0 0

Ranunculus-alejandrei_1109502_to_envalirensis 344164 339848 98.7 659 636 636 612 529 410 0 7 9 215 421 0 0

Ranunculus-alejandrei_1109502_to_flabellifolius 286862 284000 99.0 657 573 573 531 419 233 0 4 5 245 328 0 0

Ranunculus-alejandrei_1109502_to_marsicus 322358 318644 98.8 658 610 610 579 488 315 0 5 13 270 340 0 0

Ranunculus-alejandrei_1109502_to_notabilis 287615 284133 98.8 659 593 593 563 444 250 0 1 1 255 338 0 0

Ranunculus-alejandrei_1109505_to_cassubicifolius 319525 317535 99.4 627 478 478 398 210 94 0 1 1 350 128 0 0

Ranunculus-alejandrei_1109505_to_envalirensis 542271 536387 98.9 659 643 643 629 560 436 0 10 15 210 433 0 0

Ranunculus-alejandrei_1109505_to_flabellifolius 452623 448610 99.1 655 598 598 554 444 268 0 5 7 247 351 0 0

Ranunculus-alejandrei_1109505_to_marsicus 506618 501655 99.0 658 629 629 598 521 363 0 10 14 268 361 0 0

Ranunculus-alejandrei_1109505_to_notabilis 455743 450852 98.9 659 616 616 585 474 298 0 5 7 242 374 0 0

Ranunculus-alejandrei_1109603_to_cassubicifolius 213385 211928 99.3 628 458 458 377 192 76 0 2 3 349 109 0 0

Ranunculus-alejandrei_1109603_to_envalirensis 357114 353363 98.9 658 623 623 603 516 378 0 6 7 224 399 0 0

Ranunculus-alejandrei_1109603_to_flabellifolius 299770 297060 99.1 655 570 570 530 397 227 0 3 4 257 313 0 0

Ranunculus-alejandrei_1109603_to_marsicus 334964 331729 99.0 658 613 613 583 480 308 0 6 7 287 326 0 0

Ranunculus-alejandrei_1109603_to_notabilis 301620 298484 99.0 658 585 585 549 423 240 0 4 6 244 341 0 0

Ranunculus-alejandrei_1109607_to_cassubicifolius 214771 213079 99.2 627 462 462 388 201 84 0 1 1 347 115 0 0

Ranunculus-alejandrei_1109607_to_envalirensis 369906 364767 98.6 659 634 634 617 540 409 0 11 14 213 421 0 0

Ranunculus-alejandrei_1109607_to_flabellifolius 306576 303109 98.9 656 592 592 552 430 252 0 4 8 261 331 0 0

Ranunculus-alejandrei_1109607_to_marsicus 345522 341202 98.7 658 616 616 595 503 339 0 5 11 259 357 0 0

Ranunculus-alejandrei_1109607_to_notabilis 308285 304154 98.7 659 595 595 568 447 270 0 3 5 249 346 0 0

Ranunculus-aragonensis_Du3285503_to_cassubicifolius 270375 269215 99.6 630 452 452 355 159 50 0 1 1 389 63 0 0

Ranunculus-aragonensis_Du3285503_to_envalirensis 422919 420860 99.5 660 607 607 552 393 189 0 5 5 362 245 0 0

Ranunculus-aragonensis_Du3285503_to_flabellifolius 374801 373151 99.6 658 545 545 471 275 104 0 2 4 360 185 0 0

Ranunculus-aragonensis_Du3285503_to_marsicus 406878 405056 99.6 658 588 588 512 353 176 0 4 6 391 197 0 0

Ranunculus-aragonensis_Du3285503_to_notabilis 372065 370299 99.5 660 554 554 476 277 122 0 2 2 358 196 0 0

Ranunculus-baldensis_Du20887015_to_cassubicifolius 234183 232986 99.5 630 439 438 333 136 45 0 0 0 382 56 0 0

Ranunculus-baldensis_Du20887015_to_envalirensis 322604 320999 99.5 659 527 527 423 227 81 0 2 2 363 164 0 0

Ranunculus-baldensis_Du20887015_to_flabellifolius 325998 324328 99.5 656 512 512 414 218 81 0 2 2 379 133 0 0

Ranunculus-baldensis_Du20887015_to_marsicus 355617 353853 99.5 658 541 541 453 284 137 0 2 2 355 186 0 0

Ranunculus-baldensis_Du20887015_to_notabilis 330832 329131 99.5 661 530 530 436 241 91 0 1 1 373 157 0 0

Ranunculus-bovioi_Du25388010_to_cassubicifolius 293410 292044 99.5 630 461 461 365 168 65 0 1 1 382 79 0 0

Ranunculus-bovioi_Du25388010_to_envalirensis 447206 444875 99.5 659 610 609 544 391 205 0 5 5 368 241 0 0

Ranunculus-bovioi_Du25388010_to_flabellifolius 408119 406120 99.5 657 562 560 487 292 125 0 1 2 378 182 0 0

Ranunculus-bovioi_Du25388010_to_marsicus 445043 442881 99.5 658 597 597 531 362 188 0 2 4 381 216 0 0

Ranunculus-bovioi_Du25388010_to_notabilis 412656 410533 99.5 660 581 581 497 315 141 0 2 2 369 212 0 0

Ranunculus-camerinus_1109904_to_cassubicifolius 274297 272653 99.4 629 452 452 378 194 84 0 2 2 342 110 0 0

Ranunculus-camerinus_1109904_to_envalirensis 449363 445687 99.2 659 635 635 612 531 378 0 6 7 238 397 0 0

Ranunculus-camerinus_1109904_to_flabellifolius 377068 374316 99.3 658 577 577 536 395 218 0 4 9 273 304 0 0

Ranunculus-camerinus_1109904_to_marsicus 421836 418782 99.3 657 605 605 573 464 283 0 7 13 276 329 0 0

Ranunculus-camerinus_1109904_to_notabilis 375324 372405 99.2 659 588 588 550 421 234 0 2 3 268 320 0 0

Ranunculus-camerinus_1109906_to_cassubicifolius 230595 229100 99.4 629 460 459 380 206 80 0 1 1 341 118 0 0

Ranunculus-camerinus_1109906_to_envalirensis 382573 378732 99.0 659 636 636 610 541 395 0 7 9 236 400 0 0

Ranunculus-camerinus_1109906_to_flabellifolius 320257 317481 99.1 658 580 579 529 408 229 0 3 8 260 319 0 0

Ranunculus-camerinus_1109906_to_marsicus 359868 356498 99.1 658 606 606 573 475 317 0 7 13 266 340 0 0

Ranunculus-camerinus_1109906_to_notabilis 320352 317168 99.0 660 597 597 560 441 246 0 1 2 255 342 0 0

Ranunculus-camerinus_1110406_to_cassubicifolius 284923 282923 99.3 629 460 460 390 203 82 0 2 3 344 116 0 0

Ranunculus-camerinus_1110406_to_envalirensis 462143 457876 99.1 659 634 634 615 526 376 0 6 7 220 414 0 0

Ranunculus-camerinus_1110406_to_flabellifolius 400593 397152 99.1 658 589 588 543 400 232 0 5 7 302 286 0 0

Ranunculus-camerinus_1110406_to_marsicus 444565 440722 99.1 658 619 619 585 485 315 0 6 10 278 341 0 0

Ranunculus-camerinus_1110406_to_notabilis 399750 396115 99.1 660 608 608 573 435 254 0 3 4 283 325 0 0

Ranunculus-camerinus_1110409_to_cassubicifolius 291564 289576 99.3 631 464 464 388 208 83 0 5 6 347 117 0 0

Ranunculus-camerinus_1110409_to_envalirensis 473226 468992 99.1 659 635 635 615 529 381 0 6 6 225 410 0 0

Ranunculus-camerinus_1110409_to_flabellifolius 410150 406779 99.2 658 584 584 541 401 250 0 6 10 276 308 0 0

Ranunculus-camerinus_1110409_to_marsicus 456485 452710 99.2 658 623 623 591 493 323 0 8 10 287 336 0 0

Ranunculus-camerinus_1110409_to_notabilis 410311 406660 99.1 660 602 602 565 447 262 0 5 6 268 334 0 0

Ranunculus-camerinus_1110607_to_cassubicifolius 211724 210170 99.3 631 448 448 357 169 69 0 1 1 366 82 0 0

Ranunculus-camerinus_1110607_to_envalirensis 338483 335386 99.1 659 614 614 562 428 272 0 6 6 329 285 0 0

Ranunculus-camerinus_1110607_to_flabellifolius 293545 291008 99.1 658 556 556 487 308 151 0 3 4 346 210 0 0

Ranunculus-camerinus_1110607_to_marsicus 321257 318495 99.1 658 594 594 529 385 214 0 4 5 364 230 0 0

Ranunculus-camerinus_1110607_to_notabilis 292960 290379 99.1 660 568 568 503 319 147 0 2 3 346 222 0 0

Ranunculus-camerinus_1110609_to_cassubicifolius 167128 164997 98.7 631 438 438 350 137 55 0 1 1 337 101 0 0

Ranunculus-camerinus_1110609_to_envalirensis 258251 255074 98.8 659 483 483 438 291 148 0 0 0 274 209 0 0

Ranunculus-camerinus_1110609_to_flabellifolius 230946 228133 98.8 657 461 461 384 214 95 0 0 0 289 172 0 0

Ranunculus-camerinus_1110609_to_marsicus 246673 243679 98.8 658 486 485 419 272 141 0 0 1 281 204 0 0

Ranunculus-camerinus_1110609_to_notabilis 230225 227337 98.7 659 460 460 387 228 90 0 0 1 278 182 0 0

Ranunculus-cantabricus_Du2939402_to_cassubicifolius 356424 354770 99.5 630 475 475 386 181 64 0 2 2 394 81 0 0

Ranunculus-cantabricus_Du2939402_to_envalirensis 575128 571639 99.4 659 630 630 588 444 253 0 5 6 337 293 0 0

Ranunculus-cantabricus_Du2939402_to_flabellifolius 498617 495792 99.4 658 579 579 511 307 141 0 2 4 362 217 0 0

Ranunculus-cantabricus_Du2939402_to_marsicus 547894 544805 99.4 658 604 604 552 395 226 0 6 9 346 258 0 0

Ranunculus-cantabricus_Du2939402_to_notabilis 496098 493214 99.4 660 587 587 508 313 149 0 3 3 375 212 0 0

Ranunculus-carlittensis_Du2998602_to_cassubicifolius 258097 256953 99.6 632 449 449 360 159 57 0 0 0 378 71 0 0

Ranunculus-carlittensis_Du2998602_to_envalirensis 427311 425192 99.5 659 626 626 570 409 232 0 2 2 375 251 0 0

Ranunculus-carlittensis_Du2998602_to_flabellifolius 359202 357457 99.5 656 550 550 457 257 110 0 2 3 376 174 0 0

Ranunculus-carlittensis_Du2998602_to_marsicus 388311 386427 99.5 658 586 586 514 316 153 0 3 4 397 189 0 0

Ranunculus-carlittensis_Du2998602_to_notabilis 360011 358292 99.5 660 555 554 468 273 126 0 1 1 370 184 0 0

Ranunculus-cassubicifolius_9126S02 1488270 822866 55.3 683 635 633 576 435 264 0 6 7 337 296 0 0

Ranunculus-cassubicifolius_DU013S01 1303340 960900 73.7 678 663 661 651 631 618 0 33 84 214 447 0 0

Ranunculus-cassubicifolius_LH006S17 1211924 821255 67.8 680 663 661 647 632 619 0 33 69 202 459 0 0

Ranunculus-cassubicifolius_LH016S14 1856820 1292641 69.6 685 664 662 649 632 617 0 30 71 210 452 0 0

Ranunculus-cassubicifolius_LH08S07 1255598 850809 67.8 684 663 663 646 627 614 1 47 109 192 471 0 0

Ranunculus-cassubicifolius_LH09S03 1001666 682904 68.2 682 661 661 646 628 608 0 32 81 196 465 0 0

Ranunculus-cassubicifolius_LH40S04 993306 649379 65.4 684 661 660 647 627 614 1 30 64 187 473 0 0

Ranunculus-envalirensis_DU018S01 803072 553514 68.9 678 660 660 647 629 612 0 31 66 214 446 0 0

Ranunculus-envalirensis_Du019 951898 515528 54.2 686 663 663 647 629 611 0 32 55 198 465 0 0

Ranunculus-envalirensis_Du33354S02 1277390 777754 60.9 679 663 663 647 628 596 0 27 55 190 473 0 0

Ranunculus-flabellifolius_DU021S01 1321328 935039 70.8 675 663 662 647 630 614 0 34 71 193 469 0 0

Ranunculus-flabellifolius_DU054S01 942820 693809 73.6 680 662 662 651 632 617 0 40 81 221 441 0 0

Ranunculus-flabellifolius_LH25S01 897624 563184 62.7 675 661 661 647 631 607 0 27 50 198 463 0 0

Ranunculus-gortanii_BB5_to_cassubicifolius 313726 312514 99.6 631 469 469 394 211 80 0 1 3 345 124 0 0

Ranunculus-gortanii_BB5_to_envalirensis 440769 437863 99.3 659 628 628 590 477 292 0 6 7 250 378 0 0

Ranunculus-gortanii_BB5_to_flabellifolius 440968 438527 99.4 657 612 611 577 461 269 0 7 10 270 341 0 0

Ranunculus-gortanii_BB5_to_marsicus 502106 498953 99.4 658 635 635 610 526 367 0 5 8 268 367 0 0

Ranunculus-gortanii_BB5_to_notabilis 470674 467630 99.4 659 627 627 600 520 354 0 8 10 235 392 0 0

Ranunculus-gortanii_CdR3_to_cassubicifolius 305186 303991 99.6 630 473 473 404 202 74 0 3 5 368 105 0 0

Ranunculus-gortanii_CdR3_to_envalirensis 426286 423678 99.4 659 606 606 549 416 237 0 5 7 278 328 0 0

Ranunculus-gortanii_CdR3_to_flabellifolius 427771 425492 99.5 658 603 603 556 435 228 0 6 9 311 292 0 0

Ranunculus-gortanii_CdR3_to_marsicus 481141 478529 99.5 658 624 624 591 482 315 0 5 8 293 331 0 0

Ranunculus-gortanii_CdR3_to_notabilis 453052 450384 99.4 659 622 622 589 476 309 0 5 7 254 368 0 0

Ranunculus-gortanii_Du3413703_to_cassubicifolius 328661 327032 99.5 632 489 489 398 189 70 0 2 3 398 91 0 0

Ranunculus-gortanii_Du3413703_to_envalirensis 452416 449639 99.4 659 608 608 548 357 147 0 3 3 358 250 0 0

Ranunculus-gortanii_Du3413703_to_flabellifolius 453469 450924 99.4 658 588 588 526 329 156 0 2 4 359 229 0 0

Ranunculus-gortanii_Du3413703_to_marsicus 505532 502722 99.4 658 624 624 579 416 249 0 4 6 343 281 0 0

Ranunculus-gortanii_Du3413703_to_notabilis 472080 469352 99.4 660 604 604 563 394 205 0 3 3 327 277 0 0

Ranunculus-gortanii_Du3528108_to_cassubicifolius 280469 279171 99.5 632 472 472 394 193 80 0 3 5 362 110 0 0

Ranunculus-gortanii_Du3528108_to_envalirensis 383696 381006 99.3 659 594 594 541 374 194 0 4 4 311 283 0 0

Ranunculus-gortanii_Du3528108_to_flabellifolius 385506 383157 99.4 658 589 589 540 391 208 0 5 8 314 275 0 0

Ranunculus-gortanii_Du3528108_to_marsicus 433994 431215 99.4 658 622 622 582 462 292 0 7 12 318 304 0 0

Ranunculus-gortanii_Du3528108_to_notabilis 402922 400158 99.3 660 609 609 568 427 242 0 4 6 311 298 0 0

Ranunculus-guarensis_Du29560010_to_cassubicifolius 296454 295150 99.6 631 445 445 360 160 57 0 2 2 369 76 0 0

Ranunculus-guarensis_Du29560010_to_envalirensis 478845 476290 99.5 659 623 623 570 417 235 0 6 6 356 267 0 0

Ranunculus-guarensis_Du29560010_to_flabellifolius 421047 418946 99.5 657 560 560 486 289 121 0 5 6 368 192 0 0

Ranunculus-guarensis_Du29560010_to_marsicus 454288 452022 99.5 658 593 593 529 358 177 0 4 6 367 226 0 0

Ranunculus-guarensis_Du29560010_to_notabilis 412195 410095 99.5 660 566 566 487 293 130 0 4 4 355 211 0 0

Ranunculus-marsicus_LH017S01 906332 672415 74.2 677 659 659 647 631 610 0 43 92 221 438 0 0

Ranunculus-marsicus_LH018S02 1212884 847517 69.9 677 663 661 648 631 614 0 45 106 210 451 0 0

Ranunculus-notabilis_10137S03 1172086 919057 78.4 679 662 662 648 632 616 0 37 74 208 454 0 0

Ranunculus-notabilis_2 991036 263526 26.6 688 652 652 638 614 569 1 27 46 192 460 0 0

Ranunculus-notabilis_DU003S01 762410 607301 79.7 677 660 660 648 630 607 1 37 78 218 442 0 0

Ranunculus-notabilis_DU049S01 1140030 898036 78.8 682 665 663 650 630 616 1 38 86 213 450 0 0

Ranunculus-notabilis_Du35351S15 1547866 913260 59.0 680 662 661 645 625 594 0 24 53 187 474 0 0

Ranunculus-notabilis_Hoe5615 1143234 730324 63.9 684 661 660 643 615 563 1 21 42 207 453 0 0

Ranunculus-notabilis_LH010S07 734346 549065 74.8 677 658 658 645 629 611 1 39 76 212 446 0 0

Ranunculus-notabilis_LH011S01 1131922 839111 74.1 680 664 662 647 630 617 0 36 78 212 450 0 0

Ranunculus-notabilis_LH011S14 849882 637957 75.1 677 664 662 648 629 615 1 38 75 225 437 0 0

Ranunculus-notabilis_LH012S08 1048068 852072 81.3 677 662 662 651 633 616 0 33 75 212 450 0 0

Ranunculus-notabilis_LH013S03 846348 685777 81.0 678 661 661 648 631 614 1 33 73 203 458 0 0

Ranunculus-notabilis_LH014S03 1170400 943075 80.6 678 662 662 647 631 617 2 32 71 206 456 0 0

Ranunculus-notabilis_LH015S10 1283046 1032560 80.5 680 665 663 649 631 615 1 33 75 202 461 0 0

Ranunculus-notabilis_LH028S02 1831010 1317240 71.9 685 663 662 648 624 597 0 24 65 182 480 0 0

Ranunculus-palaeoeuganeus_AD1_to_cassubicifolius 223332 222487 99.6 629 465 465 388 188 72 0 0 1 373 92 0 0

Ranunculus-palaeoeuganeus_AD1_to_envalirensis 311261 309426 99.4 659 593 593 533 367 176 0 7 7 324 269 0 0

Ranunculus-palaeoeuganeus_AD1_to_flabellifolius 310702 309120 99.5 658 581 581 536 366 195 0 5 6 325 256 0 0

Ranunculus-palaeoeuganeus_AD1_to_marsicus 350197 348286 99.5 658 613 613 574 447 275 0 5 8 317 296 0 0

Ranunculus-palaeoeuganeus_AD1_to_notabilis 327248 325372 99.4 659 597 597 553 406 228 0 5 6 309 288 0 0

Ranunculus-palaeoeuganeus_Du2099804_to_cassubicifolius 237395 236376 99.6 632 460 460 381 189 70 0 1 2 369 91 0 0

Ranunculus-palaeoeuganeus_Du2099804_to_envalirensis 331613 329637 99.4 659 586 586 513 353 156 0 3 3 331 255 0 0

Ranunculus-palaeoeuganeus_Du2099804_to_flabellifolius 331482 329706 99.5 657 579 579 500 320 155 0 5 8 359 220 0 0

Ranunculus-palaeoeuganeus_Du2099804_to_marsicus 369954 367949 99.5 658 605 605 552 419 246 0 3 8 328 277 0 0

Ranunculus-palaeoeuganeus_Du2099804_to_notabilis 346916 344991 99.4 660 597 597 540 365 194 0 1 2 328 269 0 0

Ranunculus-palaeoeuganeus_Q1_to_cassubicifolius 259014 257935 99.6 631 510 510 435 230 96 0 1 2 419 91 0 0

Ranunculus-palaeoeuganeus_Q1_to_envalirensis 332643 330788 99.4 659 582 581 528 340 151 0 4 6 334 247 0 0

Ranunculus-palaeoeuganeus_Q1_to_flabellifolius 337770 336065 99.5 657 582 582 516 336 163 0 6 8 358 224 0 0

Ranunculus-palaeoeuganeus_Q1_to_marsicus 372064 370047 99.5 658 598 598 548 412 227 0 2 3 338 260 0 0

Ranunculus-palaeoeuganeus_Q1_to_notabilis 346882 344919 99.4 659 588 587 525 349 173 0 2 3 359 228 0 0

Ranunculus-palaeoeuganeus_Q2_to_cassubicifolius 331116 330025 99.7 631 522 522 453 271 114 0 2 5 379 143 0 0

Ranunculus-palaeoeuganeus_Q2_to_envalirensis 423916 421324 99.4 659 613 613 573 455 283 0 9 9 258 355 0 0

Ranunculus-palaeoeuganeus_Q2_to_flabellifolius 433642 431461 99.5 658 613 613 573 463 277 0 5 7 265 348 0 0

Ranunculus-palaeoeuganeus_Q2_to_marsicus 486351 483684 99.5 658 632 632 607 523 358 0 7 12 269 363 0 0

Ranunculus-palaeoeuganeus_Q2_to_notabilis 445677 443038 99.4 660 629 629 598 506 324 0 6 10 245 384 0 0

Ranunculus-pelendenorum_1109803_to_cassubicifolius 189782 188401 99.3 630 456 456 378 201 85 0 1 3 351 105 0 0

Ranunculus-pelendenorum_1109803_to_envalirensis 308529 305332 99.0 659 622 622 594 501 352 0 7 9 249 373 0 0

Ranunculus-pelendenorum_1109803_to_flabellifolius 264075 261559 99.0 656 568 568 525 390 215 0 3 5 272 296 0 0

Ranunculus-pelendenorum_1109803_to_marsicus 296367 293488 99.0 658 597 597 562 453 298 0 6 10 278 319 0 0

Ranunculus-pelendenorum_1109803_to_notabilis 264752 261960 98.9 660 585 585 542 424 241 0 2 2 266 319 0 0

Ranunculus-pindicola_Du26708013_to_cassubicifolius 508269 505598 99.5 632 493 493 408 210 79 0 2 3 356 137 0 0

Ranunculus-pindicola_Du26708013_to_envalirensis 704862 700202 99.3 659 608 608 558 401 222 0 6 7 299 309 0 0

Ranunculus-pindicola_Du26708013_to_flabellifolius 715221 710844 99.4 658 613 613 564 421 235 0 10 11 309 304 0 0

Ranunculus-pindicola_Du26708013_to_marsicus 797677 792721 99.4 658 638 638 610 507 335 0 9 13 292 346 0 0

Ranunculus-pindicola_Du26708013_to_notabilis 739590 734809 99.4 660 625 625 588 466 274 0 8 10 294 331 0 0

Ranunculus-poldinii_C1_to_cassubicifolius 256125 255072 99.6 630 447 447 362 185 63 0 2 3 349 98 0 0

Ranunculus-poldinii_C1_to_envalirensis 357016 354720 99.4 659 601 601 538 379 200 0 5 6 319 282 0 0

Ranunculus-poldinii_C1_to_flabellifolius 358213 356297 99.5 658 591 591 523 363 191 0 5 6 337 254 0 0

Ranunculus-poldinii_C1_to_marsicus 399587 397221 99.4 658 614 614 576 429 281 0 5 10 333 281 0 0

Ranunculus-poldinii_C1_to_notabilis 377741 375443 99.4 659 607 607 564 416 243 0 4 6 311 296 0 0

Ranunculus-poldinii_CB4_to_cassubicifolius 331541 329915 99.5 632 475 475 397 219 85 0 2 4 360 115 0 0

Ranunculus-poldinii_CB4_to_envalirensis 463865 460631 99.3 659 608 608 564 442 268 0 4 6 263 345 0 0

Ranunculus-poldinii_CB4_to_flabellifolius 463970 461175 99.4 657 607 607 567 428 253 0 4 7 297 310 0 0

Ranunculus-poldinii_CB4_to_marsicus 522962 519658 99.4 658 628 628 595 505 334 0 5 11 278 350 0 0

Ranunculus-poldinii_CB4_to_notabilis 491900 488621 99.3 660 627 627 593 488 317 0 7 8 271 356 0 0

Ranunculus-pygmaeus_LG09 1591966 1302472 81.8 679 667 663 647 631 612 0 30 45 208 455 0 0

Ranunculus-rotundifrons_1110202_to_cassubicifolius 303642 301187 99.2 630 478 477 401 224 83 0 2 2 361 116 0 0

Ranunculus-rotundifrons_1110202_to_envalirensis 489410 484536 99.0 659 628 628 601 525 383 0 4 6 228 400 0 0

Ranunculus-rotundifrons_1110202_to_flabellifolius 416801 412793 99.0 658 578 578 538 424 246 0 3 8 262 316 0 0

Ranunculus-rotundifrons_1110202_to_marsicus 463522 459101 99.0 658 615 615 578 483 307 0 6 12 292 323 0 0

Ranunculus-rotundifrons_1110202_to_notabilis 417795 413578 99.0 659 601 601 571 440 247 0 2 3 263 338 0 0

Ranunculus-rotundifrons_1110205_to_cassubicifolius 240721 239221 99.4 629 455 455 370 190 77 0 2 2 354 101 0 0

Ranunculus-rotundifrons_1110205_to_envalirensis 382334 379475 99.3 659 621 621 589 493 340 0 7 8 249 372 0 0

Ranunculus-rotundifrons_1110205_to_flabellifolius 327334 324955 99.3 657 556 556 510 373 190 0 5 6 297 259 0 0

Ranunculus-rotundifrons_1110205_to_marsicus 362195 359698 99.3 658 595 595 558 448 286 0 7 9 296 299 0 0

Ranunculus-rotundifrons_1110205_to_notabilis 327071 324610 99.2 658 581 581 544 394 200 0 3 4 292 289 0 0

Ranunculus-rotundifrons_Du3283202_to_cassubicifolius 401733 399801 99.5 632 444 444 350 169 63 0 1 1 360 84 0 0

Ranunculus-rotundifrons_Du3283202_to_envalirensis 645945 642382 99.4 659 619 619 567 426 242 0 2 2 336 283 0 0

Ranunculus-rotundifrons_Du3283202_to_flabellifolius 563138 560377 99.5 658 549 549 472 288 127 0 1 3 349 200 0 0

Ranunculus-rotundifrons_Du3283202_to_marsicus 604988 601966 99.5 658 599 599 529 370 195 0 2 4 352 247 0 0

Ranunculus-rotundifrons_Du3283202_to_notabilis 557037 554149 99.5 660 558 558 487 294 131 0 2 2 361 197 0 0

Ranunculus-sceleratus_10426S3 1839446 1444819 78.5 683 665 662 645 630 601 0 131 337 207 455 0 0

Ranunculus-sennianus_Du2996806_to_cassubicifolius 324869 323360 99.5 631 453 453 364 169 68 0 2 2 366 87 0 0

Ranunculus-sennianus_Du2996806_to_envalirensis 514091 511422 99.5 659 624 624 563 404 223 0 7 7 371 253 0 0

Ranunculus-sennianus_Du2996806_to_flabellifolius 453967 451762 99.5 658 562 562 477 294 124 0 1 1 376 186 0 0

Ranunculus-sennianus_Du2996806_to_marsicus 492571 490123 99.5 657 593 593 528 363 184 0 3 5 370 223 0 0

Ranunculus-sennianus_Du2996806_to_notabilis 456571 454324 99.5 660 567 567 487 304 136 0 1 1 346 221 0 0

Ranunculus-valdesii_Du292204_to_cassubicifolius 371597 369800 99.5 631 448 448 361 174 66 0 1 1 370 78 0 0

Ranunculus-valdesii_Du292204_to_envalirensis 578062 574978 99.5 659 624 624 575 419 245 0 6 6 348 276 0 0

Ranunculus-valdesii_Du292204_to_flabellifolius 513089 510497 99.5 658 575 575 495 318 128 0 2 5 390 185 0 0

Ranunculus-valdesii_Du292204_to_marsicus 559741 556906 99.5 658 610 610 552 391 212 0 5 6 367 243 0 0

Ranunculus-valdesii_Du292204_to_notabilis 517994 515196 99.5 660 591 591 528 338 146 0 3 4 355 236 0 0

Ranunculus-varsonicus_1109702_to_cassubicifolius 292436 290531 99.3 630 466 466 384 212 97 0 1 2 344 122 0 0

Ranunculus-varsonicus_1109702_to_envalirensis 485284 480918 99.1 659 639 639 620 546 415 0 7 10 215 424 0 0

Ranunculus-varsonicus_1109702_to_flabellifolius 406817 403449 99.2 658 587 587 540 430 247 0 4 9 256 331 0 0

Ranunculus-varsonicus_1109702_to_marsicus 456776 452857 99.1 658 614 614 588 488 338 0 6 14 266 348 0 0

Ranunculus-varsonicus_1109702_to_notabilis 406490 402769 99.1 659 604 604 567 459 269 0 4 5 255 349 0 0

Ranunculus-varsonicus_1109703_to_cassubicifolius 248664 246736 99.2 628 462 462 386 208 83 0 1 2 338 124 0 0

Ranunculus-varsonicus_1109703_to_envalirensis 404220 399832 98.9 659 630 630 608 529 411 0 6 9 223 407 0 0

Ranunculus-varsonicus_1109703_to_flabellifolius 346091 342674 99.0 658 580 580 535 407 232 0 3 7 278 302 0 0

Ranunculus-varsonicus_1109703_to_marsicus 388384 384447 99.0 658 618 618 585 487 327 0 6 13 271 347 0 0

Ranunculus-varsonicus_1109703_to_notabilis 346582 342818 98.9 659 602 601 569 449 271 0 3 5 253 348 0 0

Ranunculus-vasconicus_1109203_to_cassubicifolius 404356 400411 99.0 629 492 492 412 245 110 0 5 6 348 144 0 0

Ranunculus-vasconicus_1109203_to_envalirensis 672642 662459 98.5 659 641 641 628 565 466 0 11 13 220 421 0 0

Ranunculus-vasconicus_1109203_to_flabellifolius 579647 571865 98.7 657 616 616 583 475 302 0 9 14 272 344 0 0

Ranunculus-vasconicus_1109203_to_marsicus 641897 632853 98.6 658 636 636 613 529 387 0 12 17 260 376 0 0

Ranunculus-vasconicus_1109203_to_notabilis 573828 565313 98.5 660 633 633 609 516 352 0 6 8 252 381 0 0

**Config – HybPhaser**

intronerated_contig = "yes"

# missing data

remove_samples_with_less_than_this_propotion_of_loci_recovered = 0.6

remove_samples_with_less_than_this_propotion_of_target_sequence_length_recovered = 0.6

remove_loci_with_less_than_this_propotion_of_samples_recovered = 0.75

remove_loci_with_less_than_this_propotion_of_target_sequence_length_recovered = 0.75

# Paralogs

remove_loci_for_all_samples_with_more_than_this_mean_proportion_of_SNPs = "none" # any number between 0 and 1, "none" or "outliers"

file_with_putative_paralogs_to_remove_for_all_samples = ""

remove_outlier_loci_for_each_sample = "no"

**References**

See main text for references given here.
