# Supplementary figures and images for: Phylogenomics of Southern European Taxa in the Ranunculus auricomus Species Complex: The Apple Doesn’t Fall Far from the Tree
Source: Plants (Basel). 2023 Oct 24;12(21):3664. doi: 10.3390/plants12213664 (PMC10650656; doi:10.3390/plants12213664)

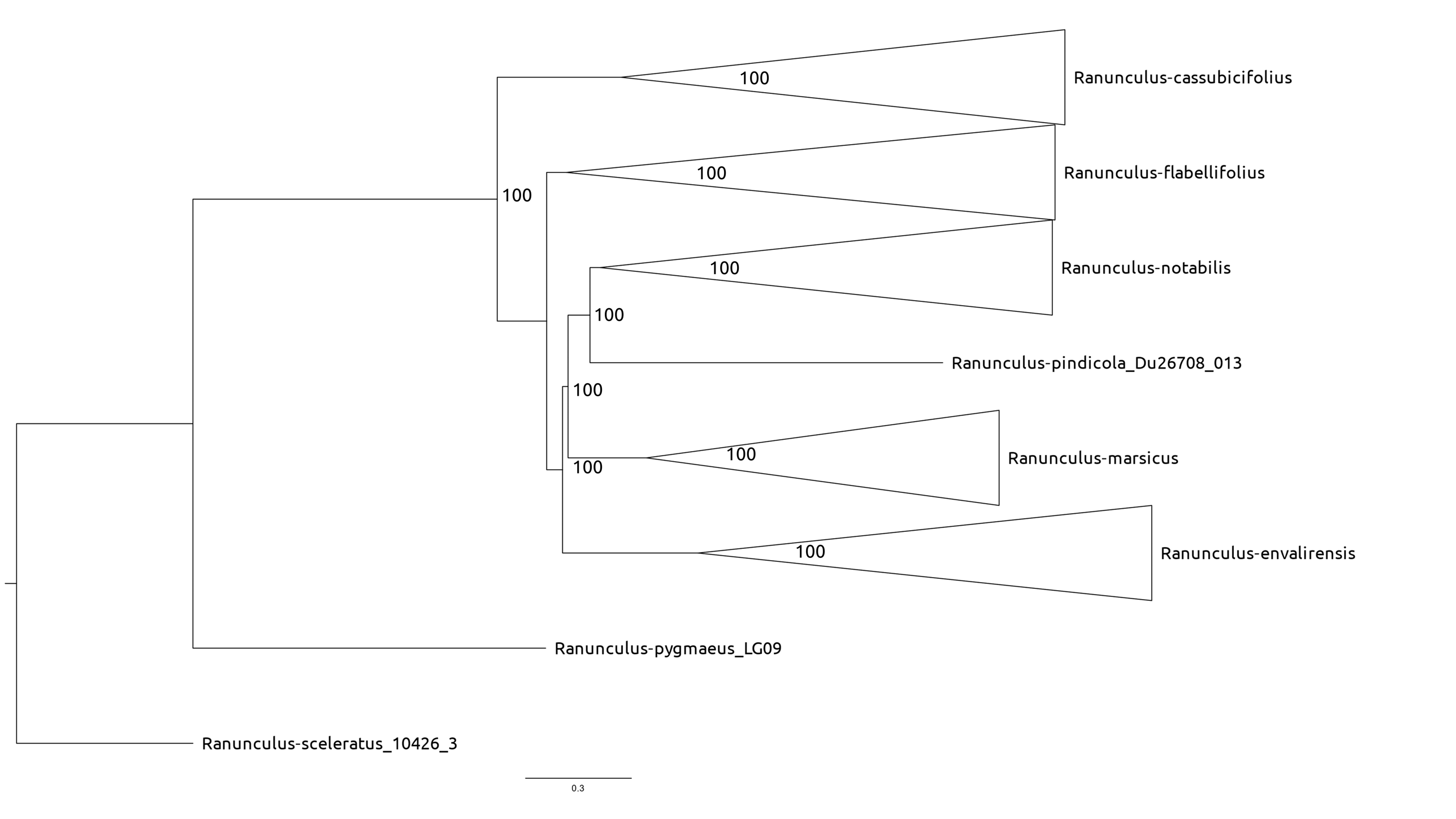

Supplement: Supplementary file 1 [file plants-12-03664-s001.zip › Figure_S1.png]
